# The prevalence and associated factors for primary headache disorders in adolescents in eastern Sudan: a community-based cross-sectional study

**DOI:** 10.3389/fneur.2024.1373890

**Published:** 2024-04-17

**Authors:** Saeed M. Omar, Osman M. Osman, Abdullah Al-Nafeesah, Ashwaq AlEed, Jaber Alfaifi, Ishag Adam

**Affiliations:** ^1^Faculty of Medicine, Gadarif University, Gadarif, Sudan; ^2^Department of Pediatrics, College of Medicine, Qassim University, Buraydah, Saudi Arabia; ^3^Department of Child Health, College of Medicine, University of Bisha, Bisha, Saudi Arabia; ^4^Department of Obstetrics and Gynecology, College of Medicine, Qassim University, Buraydah, Saudi Arabia

**Keywords:** adolescent, female, male, headache, age, migraine

## Abstract

**Background:**

Headache disorder is the second-highest cause of disability worldwide; however, data are scarce on headache among adolescents, especially in Africa. There has yet to be published data on headache among adolescents in Sudan, the third-largest country in Africa. This study aimed to assess the prevalence of primary headache disorders and associated factors among adolescents (10–19 years) in eastern Sudan.

**Methods:**

A community-based cross-sectional study was conducted in the city of Gadarif in eastern Sudan. Questionnaires were used to gather the adolescents’ sociodemographic characteristics. Headache diagnostic questions were based on the beta version of the International Classification of Headache Disorders-III (ICHD-3). Multivariate analysis was conducted to assess the associated factors for primary headache disorders, and the results were expressed as risk ratios (RRs) and 95.0% confidence interval (CI).

**Results:**

Of the 401 enrolled adolescents, 186 (46.4%) and 215 (53.6%) were male and female, respectively. The median (IQR) age was 14.0 (12.1–16.2) years. Eighty-one (20.2%) of the 401 adolescents reported experiencing primary headache disorders, including migraine with aura in 16 (4.0%), migraine without aura in 33 (8.2%), tension-type in 14 (3.5%), and undifferentiated headache in 18 (4.5%) adolescents. The prevalence of primary headache disorders was significantly higher in females than in males [55/215 (67.9%) vs. 26/186 (32.1%), *p* = 0.004]. In the multivariate analysis, increased age (*RR* = 1.09, 95.0 *CI* = 1.02–1.16) and being female (*RR* = 1.75, 95.0 *CI* = 1.14–2.67) were associated with increased RR of primary headache disorders. Parents’ education level and occupation, smoking/snuff use, and body mass index were not associated with primary headache disorders.

**Conclusion:**

One-fifth of the adolescents in eastern Sudan reported experiencing primary headache disorders, which was more common in females and with increased age.

## Introduction

The World Health Organization (WHO) defines adolescence (ages 10–19) as “the phase of human life that lies between childhood and adulthood” (1). Adolescence is a key phase/stage of human development and an important period in terms of its effect on future health (1).

Recent reports in the Middle East, Asia, and Africa have shown that the prevalence of headache among adolescents is almost similar to that among adults; however, the epidemiology of headache is less investigated among adolescents than adults (2). Primary headache disorders among children and adolescents are a worldwide health problem (3, 4). According to an assessment of the global burden of disease in 2016, migraine was the most disabling disease in the 15–49 age group (5). Several factors, such as diet, dehydration, stress, hormones, medication, and genetic factors, are related to the development of primary headache disorders among children (6). Migraine is also associated with comorbidities such as allergies and sleep disorders. It has recently been shown that headache is the leading reported cause of school absenteeism among children and adolescents (7). Although the exact etiology of primary headache disorders among adolescents is not yet fully understood (8), several factors, such as age (7, 9), sex (4, 10, 11), obesity, underweight (12), anxiety/depression (11, 13, 14), and sleep disorders (11, 15) are associated with primary headache disorders among children and adolescents. Recent reports show varied primary headache disorders rates among adolescents in different countries (3, 7, 9–11, 16). There are few published data on primary headache disorders among adolescents in Africa (17–20), although high-quality primary headache disorders studies in this age group are urgently needed. No studies have been conducted in Sudan, the third-largest country in Africa. The current study assessed the prevalence and factors associated with primary headache disorders among adolescents in eastern Sudan.

## Methods

### Sampling and recruitment

The Strengthening the Reporting of Observational Studies in Epidemiology (STROBE) Statement standard checklists were followed (21). A cross-sectional community-based study was conducted in Gadarif in eastern Sudan from August to October 2023. Gadarif is the largest city in the state of Gadarif. It is also the state’s capital, one of the 18 Sudanese states on the Ethiopian border, with a population of over 1,400,000, with ethnic groups representing several tribes (22). The city is divided into four squares (Mouraba), which comprise 13 neighborhoods (Hay).

A multistage sampling study was conducted in 128 households randomly selected from the 13 neighborhoods. The desired sample size was recruited according to neighborhood size. A total sample of 401 adolescents (both male and female) with an age range of 10 – 19 was selected from households via a lottery method. If there was no adolescent in the selected household, or if they refused to participate or had met one of the exclusion criteria, the next household was chosen.

### Inclusion and exclusion criteria

The inclusion criteria were adolescents who (1) were male or female residents of the area of the study, (2) were 10–19 years of age, (3) were healthy, and (4) provided signed consent from both themselves and their guardians. The exclusion criteria included adolescents who (1) refused to consent, (2) were under 10 or over 19 years of age, (3) were pregnant, (4) were critically ill patients with severe acute illness, (5) had chronic diseases (e.g., diabetes, thyroid diseases, and heart failure), and (6) were taking medicine.

Two medical officers interviewed the eligible adolescents using questionnaires completed during face-to-face interviews. The adolescents’ age, sex, smoking and alcohol use habits, and parent’s education level and occupation were recorded. Furthermore, the adolescents’ weight and height were measured twice using standard procedures, and the mean of the two readings was used to compute their body mass index (BMI). Then, a question was introduced: “Have you had a headache that was not part of any other illness?”

The part of the questionnaires on headache was based on the headache screening and diagnostic questions in the International Classification of Headache Disorders-III (ICHD-3), beta version, criteria (23). The details of these questions are shown in Table 1. These questions covered the time, type, intensity, location, frequency, and aura symptoms. These data were then used to classify headache into the following categories: migraines without aura (“showing at least two of the following characteristics: unilateral location, pulsatile, moderate or strong intensity; and at least one of the following symptoms: nausea and vomiting and photophobia with phonophobia.”), migraines with aura (showing at least two characteristics: “unilateral location, pulsatile, moderate or strong intensity, and the report of one or more symptoms of aura that were completely reversible),” tension-type headaches [“a headache presented at least two characteristics: bilateral location, tightness or pressure (non-pulsatile), mild to moderate intensity, and no nausea or vomiting”], and undifferentiated headaches “(all the other types did not fit the previous classifications).”

**Table 1:** Questions used to collect information on primary headache disorders among adolescents in eastern Sudan (2023).

Variable	
Time	Morning, afternoon, end of the day, or at night.
Intensity	Weak, moderate, strong, or incapacitating.
Location	Left side, right side, as much on the left or as much on the right side on both side of the head, in the forehead, in the temple, at the back of the eyes, in the nape, in the neck, or other.
Type	Pressure, penetrating like a knife, thumping like a tight band around the head, burning, constant, or other.
Frequency	Daily, at least once a week, and at least once a month.
Visual	Bright lights, flashes of light, zig-zag lines, multicolored lights, and blurred vision.
Sensorial	Numbness or tingling, speech and/or language issues (aphasia).
Brainstem	Buzzing, vertigo, dysarthria, diplopia, and hypacusis.
Retinal	Partial loss of vision.
	Nausea/upset stomach, vomiting, bright lights/sun bothers you, loud sounds bother you, strong smells/odors bother you, dizziness/light headedness, vertigo, numbness or tingling, increased sensitivity of scalp/hair/ears, eye tears, runny or stuffy nose, difficulty concentrating, and mood changes/irritability.

### Sample size calculation

The sample size of 401 adolescents was computed using the OpenEpi Menu (24). Depending on the prevalence (72.8%) of primary headache disorders among adolescents in a nearby country (Ethiopia) (18) and among medical students in Sudan (43.7%) (25), we assumed that 50.0% of the adolescents would report experiencing primary headache disorders. Then, we assumed that 48.0% of the adolescents who reported primary headache disorders would be female, while 35.0% of those not reporting primary headache disorders would be female. The calculated sample size of 401 adolescents was chosen to achieve a power of 80% and a type I error rate (i.e., *p* value <0.05).

### Statistical analysis

The IBM Statistical Product and Service Solutions (SPSS) for Windows (version 22.0; SPSS Inc., New York, NY, United States) software was used to analyze the data. The Shapiro–Wilk test was used to check the normality of the continuous data. As the data did not follow a normal distribution, the median [interquartile range (IQR)] was used to express its values. A univariate analysis was initially conducted using primary headache disorders as the dependent variable, and age, sex, BMI, parental educational level, occupation, and smoking/snuff use were independent variables. Variables with a *p* value of <0.20 were shifted to build a multivariable logistic regression model to adjust for covariates. The results of adjusted risk ratios (ARRs) with their 95% confidence intervals (CIs) were calculated, and a *p* value of <0.05 was considered statistically significant.

## Results

### General characteristics

Four hundred and one adolescents were enrolled in the study; 186 (46.4%) and 215 (53.6%) were male and female, respectively. The median (IQR) ages and BMIs were 14.0 (12.1–16.2) years and 16.9 (15.2–20.2) kg/m^2^, respectively. Of the 401 adolescents, 280 (69.8%) had mothers with education at or beyond the secondary level, and 302 (75.3%) had fathers with education at or beyond the secondary level. Only 73 (18.2%) of the adolescents’ mothers were employed. Of the 401 adolescents, 8 (2.0%) were smokers/snuff users, and none were alcohol users (Table 2).

**Table 2:** Characteristics of the studied adolescents and univariate analysis of primary headache disorders in eastern Sudan (*n* = 401) (2023).

Variable		Total (*n* = 401)	Adolescents with primary headache disorders (81/401 = 20.2%)	Adolescents without primary headache disorders (320/384 = 79.8%)	Unadjusted risk ratios (95% confidence interval)	*p* value
		Median (Interquartile range)				
Age, years		14.0 (12.1–16.2)	15.2 (12.6–17.0)	13.7 (11.9–16.0)	1.10 (1.02–1.18)	0.006
Body mass index, kg/m^2^		16.9 (15.2–20.2)	17.4 (15.5–21.11)	16.8 (15.1–19.8)	1.01 (0.97–1.04)	0.513
		Frequency (proportion)				
Gender	Male	186 (46.4)	26 (32.1)	160 (50.0)	Reference	
	Female	215 (53.6)	55 (67.9)	160 (50.0)	1.16 (1.05–1.28)	0.004
Mother’s education level	≥ Secondary	280 (69.8)	60 (74.1)	220 (68.8)	Reference	
	< Secondary	121 (30.2)	21 (25.9)	100 (31.3)	0.95 (0.85–1.05)	0.331
Mother’s occupation	Housewife	328 (81.8)	67 (82.7)	261 (81.6)	Reference	
	Employed	73 (18.2)	14 (17.3)	59 (18.4)	0.98 (0.86–1.11)	0.827
Father’s education	≥ Secondary	302 (75.3)	64 (79.0)	238 (74.4)	Reference	
	< Secondary	99 (24.7)	17 (21.0)	82 (25.6)	0.95 (0.85–1.05)	0.465
Father’s occupation	Skilled	159 (39.7)	28 (24.6)	131 (40.9)	Reference	
	Labor	242 (60.3)	53 (65.4)	189 (59.1)	0.94 (0.85–1.04)	0.507
Smoking/snuff	No	393 (98.0)	81 (100.0)	312 (97.5)	Reference	0.323
	Yes	8 (2.0)	0 (0.0)	8 (2.5)	0.79 (0.76–0.84)	

### Characteristics of headache

Table 3 details the characteristics of primary headache disorders among adolescents. Of 81 adolescents reporting primary headache disorders, 69.1% had primary headache disorders at least once per week, 51.9% of the primary headache disorders were pressure type, and 64.2% had temporal headache.

**Table 3:** Headache characteristics among adolescents in eastern Sudan (2023).

Variable		Number (81)	Percentage (100)
Time of onset	Morning	19	23.5
	Afternoon	22	27.2
	End of the day	9	11.1
	At night	31	38.3
Intensity	Mild	9	11.1
	Moderate	42	51.9
	Strong/incapacitating	30	37.0
Location	Temporal	52	64.2
	Frontal	9	11.1
	Bilateral (both sides)	14	17.3
	Occipital	1	1.2
	Back of the eyes	4	4.9
	Other	1	1.2
Type of pain	Pulsatile	14	17.3
	Pressure	42	51.9
	Constant	2	2.5
	Penetrating	21	25.9
	Others	2	2.5
Frequency of pain	Daily	15	18.5
	At least once a week	56	69.1
	At least once a month	10	12.3

### Prevalence and types of headaches

Eighty-one (20.2%) of the 401 adolescents had primary headache disorders, including migraine with aura in 16 (4.0%), migraine without aura in 33 (8.2%), tension-type in 14 (3.5%), and undifferentiated headache in 18 (4.5%). The prevalence of headache was significantly higher in females compared with males [55/215 (67.9%) vs. 26/186 (32.1%), *p* = 0.004]. Of the 81 who reported experiencing primary headache disorders, 50 (61.7%) and 31 (38.3%) reported migraine headaches and non-migraine headaches, respectively. There was no significant difference with respect to age [15.1 (13.1–17.1) years vs. 15.5 (12.3–16.8) years, *p* = 0.627] or sex [17/26 (65.4%) of males vs. 33/55 (60.0%) of females had migraine headaches, *p* = 0.807] among adolescents with migraine and non-migraine headaches.

### Factors associated with headache

In univariate analysis, while increasing age (*RR* = 1.10, 95.0 *CI* = 1.02–1.18) and being female [*RR* = 1.01, 95.0 *CI* = (0.97–1.04)] were associated with increased RR of primary headache disorders, parents’ education, and occupation or smoking/snuff were not associated with primary headache disorders (Table 2). After adjustment, increased age (*RR* = 1.09, 95.0 *CI* = 1.02–1.16, *p* = 0.015) and being female (*RR* = 1.75, 95.0 *CI* = 1.14–2.67, *p* = 0.010) were associated with increased risk ratios of primary headache disorders in multivariate analysis.

## Discussion

The main findings of the current study are as follows. Of the 401 adolescents, 20.2% reported experiencing primary headache disorders. The primary headache disorders were further classified as migraine without aura (8.2%), migraine with aura (4.0%), tension-type (3.5%), and undifferentiated headache (4.5%). The current result for the prevalence of primary headache disorders among adolescents was lower than others recently reported. For example, 43.7% of the students at the University of Khartoum in Sudan (mean age 19.2 years) reported having at least two headaches in the previous 3 months (25). The prevalence of primary headache disorders (20.2%) among adolescents in the current study was lower than among adolescents in Ethiopia (72.8%) in total, further categorized as migraine (38.6%), tension-type (19.9%), and undifferentiated (12.3%) (18). Moreover, our results for adolescents who reported primary headache disorders (20.2%) were lower than the prevalence (85.8%) of primary headache disorders found among children and adolescents in Zambia, of whom 53.2% reported migraines, 12.1% tension-type headaches, and 14.8% undifferentiated headache (17). Furthermore, the prevalence (20.2%) of primary headache disorders observed in the current study was slightly lower than the prevalence (23.7%) reported among 1,500 students (mean age 20.9 years) in Nigeria (26). Furthermore, our results for the prevalence of primary headache disorders among adolescents were lower than among adolescents in several other countries, including Jordan (46.6%) (27), Kuwait (42.7%) (9), Turkey (56.4%) (7), and Lithuania (76.6%) (16). In a systematic review that included 48 studies among children and adolescents, the pooled prevalence of primary headache disorders was 62% (11% migraine, 17% tension-type), with 38 and 27% of females and males reporting headaches, respectively (3). However, our results on the prevalence of primary headache disorders contrasted with those reported by Pothmann et al. (28) and Wöber-Bingöl (29), who reported more than twice what is reported. However, the prevalence of migraine is similar in their works and our findings (28, 29). The summary of the previous studies on the prevalence of primary headache disorders is shown in Table 4.

**Table 4:** Results of the previous studies of primary headache disorders in different countries.

References	Location	Percentage of primary headache disorders	Percentage of migraine	Percentage tension-type headache	Percentage of undifferentiated headache
Zewde et al. (18)	Ethiopia	72.8	38.6	19.9	12.3
Kawatu et al. (17)	Zambia	85.8	53.2	12.1	14.8
Sanya et al. (26)	Nigeria	23.7	2.4	12.5	8.9
Albashtawy et al. (27)	Jordan	46.6	4.8	10.3	31.5
Shuaibi et al. (9)	Kuwait	42.7	20.7	18.8	-
Karaaslan et al. (7)	Turkey	56.4	5.2	26.1	25.2
Onofri et al. (3)	Systematic review	62.0	11.0	17.0	-

The difference in the prevalence of primary headache disorders found in the current study compared to other studies could be due to sociodemographic factors, cultural background, nutritional factors, inclusion and exclusion criteria, the enrolled participants, and the tools used to diagnose primary headache disorders. Moreover, the high variability among the prevalences compared to neighboring countries indicates the different factors associated with headache prevalence (e.g., ongoing war in the regions can lead to psychosocial stress).

The current study showed that compared to males, females were at 1.75 higher risk of experiencing primary headache disorders. This is in line with previous results. For example, a survey conducted among university students at Khartoum University in Sudan (mean age 19.2 years) revealed that females were at higher risk for migraine than males (25). In a study of Ethiopian adolescents, headache, especially the migrainous type, was found to be more prevalent in females (76.2%) than in males (71.0%) (18). In Jordan, girls aged 14–15 reported more headaches than boys (27). Moreover, headache was found to be more prevalent, and primary headaches were significantly more frequent in girls (66.46%) than boys (38.49%; *p* < 0.001) among Kuwaiti middle schoolers; however, no significant difference between genders was noted among primary school students in Kuwait (9). Among 5,944 adolescents (50.7% boys and 49.3% girls), being female was associated with an increased risk of having a headache in Turkey (7). Girls also showed a significantly higher headache frequency than boys among adolescents in Germany (10), Indonesia (11), Lithuania (except tension headache) (16), and Brazil (15). On the other hand, no such association was observed with gender among Canadian adolescents (14), and only a weak association was reported between migraine, tension headache, and female gender among adolescents in Zambia (17). The increased female**-**specific prevalence of headache suggests a role played by female sex hormones in the course of headache disorder in females (30). Headache in adolescent females is influenced by hormonal status, especially during menarche/menstruation, as well as during puberty (30).

In this study, each 1-year increase in age was associated with a 12.0 increase in primary headache disorders frequency (*ARR* = 1.09). This is in line with previous studies in Kuwait (9) and Turkey (7) showing that headache in adolescents was associated with age. Moreover, adolescents reporting tension headache were significantly older in Iran (31). However, a previous study in Ethiopia showed no association between age and headache among adolescents (18).

In the current study, BMI was not associated with primary headache disorders. A meta-analysis of eight studies, including 16,556 patients, showed no association between overweight, obesity, and migraine among adolescents (32). However, a recent study showed that both underweight and obesity were associated with migraine among adolescents (12).

## Limitations of the study

The current study was conducted in one city in eastern Sudan; hence, its results may not be generalizable to other regions of Sudan. Several factors, such as anxiety, depression (11, 13), sleep disturbance (11, 15), atopic disease, and iron-deficiency anemia (33), were reported to be associated with headache in adolescents, and we have not assessed these factors. Nor did we follow up with these adolescents; if we had, perhaps some findings could have been further investigated via MRI and other tools. For example, a previous study showed that during follow-up with adolescents with migraine and tension headache, MRI findings, including arachnoid cysts, pituitary adenoma, and pineal cysts, were observed in 7.7% of adolescents with migraine and 12.7% of adolescents with tension-type headache (34). Elements of bias exist, e.g., reporting, non-response, and recall bias. We calculated the sample size based on the assumption of the prevalence of headache among adolescents in the nearby country of Ethiopia; however, in light of our finding that only 20.0% reported experiencing headache, the power of the study has of course been influenced. Thus, a larger multicenter study is needed.

## Conclusion

One-fifth of the adolescents in eastern Sudan reported headaches, which were more common among females and with increased age.

## Data availability statement

The original contributions presented in the study are included in the article/supplementary material, further inquiries can be directed to the corresponding author.

## Ethics statement

Ethical approval was obtained from the research Ethical Committee of the Faculty of Medicine, University of Gadarif, Gadarif, Sudan (Ref. #2023, 14). The studies were conducted in accordance with the local legislation and institutional requirements. Written informed consent for participation in this study was provided by the participants’ legal guardians/next of kin.

## Author contributions

SO: Conceptualization, Data curation, Formal Analysis, Investigation, Methodology, Validation, Writing – original draft, Writing – review & editing. OO: Conceptualization, Data curation, Formal Analysis, Methodology, Writing – original draft, Writing – review & editing. AbA: Conceptualization, Data curation, Supervision, Writing – original draft, Writing – review & editing. AsA: Conceptualization, Supervision, Writing – original draft, Writing – review & editing. JA: Conceptualization, Data curation, Formal Analysis, Validation, Visualization, Writing – original draft, Writing – review & editing. IA: Conceptualization, Data curation, Formal Analysis, Methodology, Supervision, Validation, Visualization, Writing – original draft, Writing – review & editing.
